# Frequency of work-related musculoskeletal disorders among physical therapists: a systematic review

**DOI:** 10.47626/1679-4435-2025-1481

**Published:** 2025-09-29

**Authors:** Tahir Mahmood, Waqar Afzal, Wajeeha Mahmood, Umer Maqsood, Alberto Sumiya

**Affiliations:** 1 Department of Physical Therapy, Rashid Latif Khan University, Lahore, Pakistan.; 2 University Institute of Physical Therapy, The University of Lahore, Lahore, Pakistan.; 3 Department of Physical Therapy, University of Health Sciences, Lahore, Pakistan.; 4 Department of Physical Therapy, Azra Naheed Medical College, Lahore, Pakistan.; 5 Department of Biosciences and One health, Medical School, Universidade Federal de Santa Catarina, Curitibanos, SC, Brasil.

**Keywords:** health personnel, physical therapy specialty, occupational risks, occupational health, musculoskeletal diseases, pessoal de saúde, especialidade de fisioterapia, riscos ocupacionais, saúde do trabalhador, doenças musculoesqueléticas

## Abstract

Musculoskeletal disorders are common among physical therapists because of constant physical work and mechanical stress during interventions. The aim is to synthesize the information of work-related musculoskeletal disorders among physical therapists. A systematic review of observational studies was conducted on PubMed and Google Academics, considering for searching the period of 2015 and 2021. The methodological quality of the studies included were appraised using the Critical Appraisal Skills Programme. The average percentages were calculated by adding the percentages of individual studies for each region and divided by the total number of studies. A total of 74 articles was screened, and 20 of which were assessed. In general, the frequency of work-related musculoskeletal disorders was high (75.27%), with low back pain (54.97%) in first, followed by neck pain (36.16%), upper back pain (30.18%), shoulder (25.52%), hand/wrist (20.92%; 28.32%) and knees (19.08%). It was concluded that physical therapists have high prevalence of work-related musculoskeletal disorders, in which low back, neck, shoulder pain and knees were the most reported body regions.

## Abstract (pt)

Distúrbios musculoesqueléticos são comuns entre fisioterapeutas por causa do trabalho físico constante e estresse durante intervenções. O objetivo é sintetizar informações sobre desordens musculoesqueléticas relacionadas ao trabalho entre fisioterapeutas. Realizou-se uma revisão sistemática de estudos observacionais realizada por meio da PubMed e do Google Acadêmico, considerando o período de 2015 a 2021. Os estudos foram avaliados utilizando a escala Programa de habilidades de avaliação crítica (Critical Appraisal Skills Programme). A média das porcentagens foi calculada pela soma das porcentagens dos estudos individuais de cada região e, depois, dividindo-a pelo número total de estudos incluídos. Um total de 74 artigos foi encontrado, e 20 foram avaliados. Em geral, a frequência de desordens musculoesqueléticas foi alta (75,27%), com lombalgia (54,97%) em primeiro, seguida por cervicalgia (36,16%), dorsalgia (30,18%), desordens no ombro (25,52%), mão/punho (20,92%; 28,32%) e joelhos (19,08%). Esta revisão concluiu que o fisioterapeuta apresenta alta prevalência de distúrbios osteomusculares relacionados ao trabalho, em que dor lombar, pescoço, ombros e joelhos foram as regiões do corpo mais relatadas.

## INTRODUCTION

Work-related musculoskeletal disorders (WMSDs) are common among health care professionals, who are frequently exposed to physically demanding environments and excessive biomechanical stress. These conditions can lead to poor physical functioning and the development of inflammatory disorders affecting tendons, ligaments, nerves, soft tissues, and joints as well as degenerative diseases.^1^

Among health professionals, physical therapists are particularly vulnerable due to the physical demands of their practice, which often exceed those of other health-related occupations. This increased physical burden has been associated with absenteeism, reduced quality of life, and potentially slower patient recovery.^2,3^ The Global Burden of Disease study showed a prevalence of 1.71 billion for musculoskeletal disorders, with low back pain among the most prevalent.^4^ A number of studies have examined the risks, causes, prevalence, and ergonomic factors associated with such conditions.^2-11^

For physical therapists, WMSDs tend to be more prevalent during the early stages of their careers and may decrease over time as professionals adopt improved posture and ergonomic techniques.^5,6^ Understanding these trends can inform future discussions on prevention strategies and workload management. Therefore, the aim of this systematic review was to synthesize the current evidence on the prevalence and characteristics of WMSDs among physical therapists.

## METHODS

This study was registered in the International Prospective Register of Systematic Reviews (PROSPERO) under protocol code CRD42021243974. The review followed the Preferred Reporting Items for Systematic Reviews and Meta-Analyses (PRISMA) and the Cochrane Handbook for Systematic Reviews to ensure methodological rigor and transparency.

### STUDY DESIGN AND PARTICIPANTS

This systematic review included observational studies investigating WMSDs among physical therapists.

### INCLUSION CRITERIA

Studies were included if they were available in full text, written in English, and published between 2015 and May 30, 2021.

### EXCLUSION CRITERIA

Studies were excluded if they were review articles, case reports, focused on other health professionals, or addressed variables not related to the frequency, risk factors, or prevention of WMSDs.

### SEARCH STRATEGY

A total of two databases — Google Scholar and PubMed — were searched for relevant articles. Boolean operators “AND” and “OR” were applied to combine the following search terms: “risks,” “work-related musculoskeletal disorders,” “musculoskeletal disorders,” and “physical therapist.”

### SELECTION OF STUDIES

After an initial screening of titles, two independent authors applied the inclusion and exclusion criteria to the selected studies. Disagreements between the authors were resolved by a third author.

### DATA EXTRACTION

The following data were extracted from the included studies: country of study, participants’ age, year of publication, type of WMSD or discomfort, affected body region, and prevalence rate.

### ASSESSMENT OF QUALITY

Assessment of quality of included studies was performed using the Critical Appraisal Skills Programme, which consists of 10 general questions. Each question could be answered as “yes,” “cannot tell,” or “no.” A total score of up to 10 points was assigned to each study based on how many criteria were fully met.^12^ No classification system was applied based on the total score.

### QUANTITATIVE ANALYSIS

A descriptive analysis was conducted. Average prevalence rates were calculated by summing the percentages reported in individual studies for each body region and dividing by the total number of studies included in the final analysis.

## RESULTS

**Figure 1:**
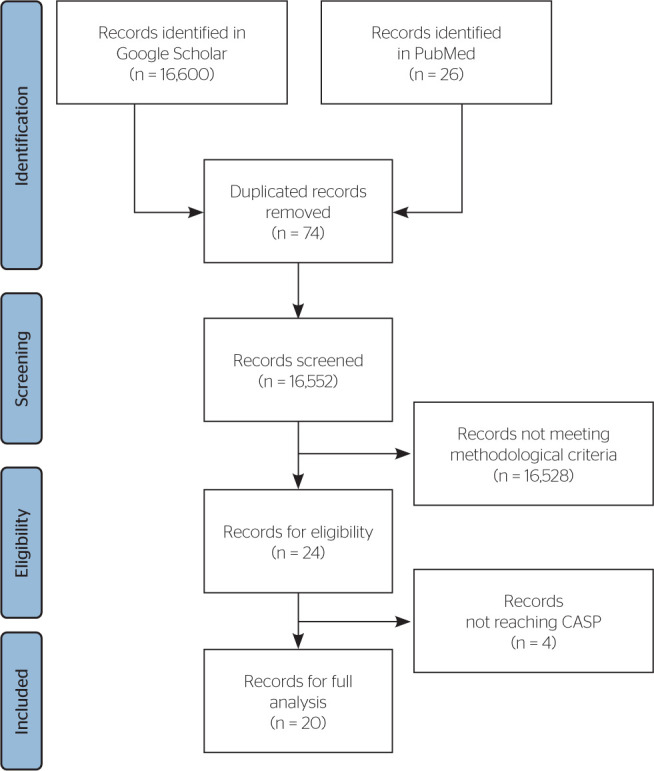
Flowchart of the study. CASP: Critical Appraisal Skills Programme.

A total of 16,626 studies were identified, of which 20 were included for full analysis after applying the inclusion and exclusion criteria (Figure 1). Data were organized into four four tables covering quality assessment, demographic characteristics, WMSDs by body region, and job-related risk factors.

### ASSESSMENT OF QUALITY

Among the 20 studies analyzed, 8 received a total score of 10 points, 7 studies scored 9 points, and 5 studies scored 8 points. The most common methodological issues were related to recruitment procedures and lack of consideration for researcher-participant dynamics. Overall, studies demonstrated good methodological quality (Table 1).

**Table 1:** Quality assessment using Critical Appraisals Skills Programme (CASP)

First author	Clear statement of research aim	Appropriate qualitative methodology	Appropriate research design	Appropriate recruitment	Data collected addressing specific issue	Researcher–participant considerations	Ethical considerations	Rigorous data analysis	Clear statement of findings	Valuable Results
Narendrasinh JU^2^	Yes	Yes	Yes	None	Yes	None	Yes	Yes	Yes	Yes
Sagahutu JB^13^	Yes	Yes	Yes	None	Yes	None	Yes	Yes	Yes	Yes
Anyfantis I^8^	Yes	Yes	Yes	None	Yes	Yes	Yes	Yes	Yes	Yes
Balakrishnan R^10^	Yes	Yes	Yes	Yes	Yes	None	Yes	Yes	Yes	Yes
Kutty RK^11^	Yes	Yes	Yes	None	Yes	None	Yes	Yes	Yes	Yes
Prerana S^14^	Yes	Yes	Yes	None	Yes	None	Yes	Yes	Yes	Yes
Pfumojena CC^15^	Yes	Yes	Yes	None	Yes	Yes	Yes	Yes	Yes	Yes
Mondal A^16^	Yes	Yes	Yes	Yes	Yes	None	Yes	Yes	Yes	Yes
Nazari H^17^	Yes	Yes	Yes	Yes	Yes	None	Yes	Yes	Yes	Yes
Happy SM^18^	Yes	Yes	Yes	Yes	Yes	None	Yes	Yes	Yes	Yes
Iqbal Z^19^	Yes	Yes	Yes	Yes	Yes	None	Yes	Yes	Yes	Yes
Vieira ER^9^	Yes	Yes	Yes	Yes	Yes	Yes	Yes	Yes	Yes	Yes
Islam M^20^	Yes	Yes	Yes	Yes	Yes	Yes	Yes	Yes	Yes	Yes
Muaidi QI^21^	Yes	Yes	Yes	Yes	Yes	Yes	Yes	Yes	Yes	Yes
Abaraogu UO^22^	Yes	Yes	Yes	Yes	Yes	Yes	Yes	Yes	Yes	Yes
Alnaser MZ^23^	Yes	Yes	Yes	Yes	Yes	Yes	Yes	Yes	Yes	Yes
Rahimi F^24^	Yes	Yes	Yes	Yes	Yes	Yes	Yes	Yes	Yes	Yes
Ezzatvar Y^25^	Yes	Yes	Yes	Yes	Yes	Yes	Yes	Yes	Yes	Yes
Bae Y-H^26^	Yes	Yes	Yes	Yes	Yes	Yes	Yes	Yes	Yes	Yes
Nasir A^27^	Yes	Yes	Yes	None	Yes	Yes	Yes	Yes	Yes	Yes

### WMSDs BY GENDER

A study from Nigeria reported a WMSD prevalence of 77.8% (38 individuals) among physical therapists, with higher prevalence in men (25 [65.7%]) compared to women (13 [24.3%]).^13^ In contrast, data from Bangladesh showed women were more affected (64.4%) than men (58.8%).^28^ Another Bangladeshi study found a higher proportion of WMSDs among women (63.16%) than men (36.84%).^18^

In India, women were generally more prone to WMSDs.^2^ A study reported that even a 1 kg increase in body weight was associated with a 29% higher risk of developing musculoskeletal pain, with women being more vulnerable to spine-related issues.^11^ Low back pain (LBP) was the most prevalent WMSD among women (64.1%) compared to men (67.9%) in a sample of 160 physical therapists^14^ (Table 2).

**Table 2:** Year-wise demographic distribution of work-related musculoskeletal disorders (WMSDs)

First author	Country	Size of study sample	Total WMSDs (n [%])	WMSD by gender		Age (mean ± SD)
				Male (n [%])	Female (n [%])	
Narendrasinh JU^2^	India	60	41 (68.0)	*	*	30 ± 9
Sagahutu JB^13^	Nigeria (Kigali)	49	38 (77.0)	65.7 (25.0)	34.3 (13.0)	30.89 ± 5.7
Anyfantis I^8^	Greece	252	224 (89.0)			42.18 ± 9.2
Balakrishnan R^10^	Malaysia	70	49 (70.0)	20 (14.0)	50 (35.0)	28.02 ± 5.9
Kutty RK^11^	Kerala (India)	190	119 (67.3)	*	*	*
Prerana S^14^	South Gujarat (India)	271	170 (62.73)	31 (52.0)	69 (118.0)	25.38 ± 3.2
Pfumojena CC^15^	Zimbabwe	261	224 (86.1)	*	*	34.5 ± 9.5
Mondal A^16^	Bangladesh (Dhaka)	61	28 (46.0)	*	*	34.5 ± 14.5
Nazari H^17^	Hamedan (Iran)	45	30 (65.9)	*	*	36.49 ± 7.4
Happy SM^18^	Bangladesh	60	57 (95.0)	36.84 (21.0)	63.16 (36.0)	26.91 ± 3.5
Iqbal Z^19^	Delhi (India)	75	67(92.0)	24 (16.0)	43 (29.0)	30.00 ± 10
Vieira ER^9^	USA(Florida)	121	116 (96.0)	*	*	43 ± 12
Islam MS^20^	Bangladesh (Dhaka)	101	96 (95.0)	*	*	27.8 ± 4.5
Muaidi QI^21^	KSA (Dammam)	690	329 (47.7)	*	*	*
Abaraogu UO^22^	Nigeria	126	100 (80.0)	*	*	31.98 ± 4.9
Alnaser MZ^23^	Kuwait	312	150 (48.0)	*	*	34. 25 ± 7.2
Rahimi F^24^	Iran	319	300 (94.0)	Less	More	47.00 ± 26
Ezzatvar Y^25^	Spain	981	560 (57.0)	*	*	34.3 ± 8
Bae Y-H^26^	South Korea	788	745 (94.5)	Less	More	35.0 ± 15
Nasir A^27^	Pakistan	56	52 (93.02)			27.30 ± 5.3

WMSDs = work-related musculoskeletal disorders; SD = standard deviation.

* Missing data in the original study.

### WMSDs BY COUNTRY

High WMSD prevalence was reported in countries such as Egypt (815, 97.6%),^7^ Greece (224, 89%), and India (41, 68%).^2^ In Canada, pediatric and neurological physical therapists also showed high rates of WMSDs.^2^ In Bangladesh, intensive care unit was associated with higher WMSD prevalence than general wards.^29^ These disorders were present in both developed and underdeveloped regions, such as Kuwait (150, 48%).^3^ In Iran, physical therapists had a lower WMSD prevalence (58.3%) compared to occupational therapists (76.4%).^8,9^

### WMSDs BY YEARS OF EXPERIENCE

WMSDs were more prevalent among health professionals with fewer years of experience. The highest prevalence was found in those with 1-5 years of experience (52%), with a notable decrease among those with 6-15 years (13%). In Greece, 32.2% of physical therapists developed injuries within their first 5 years of practice.^8,10^

A 2015 study also observed that longer working hours increased the risk of WMSDs.^11^ Similarly, a study from Bangladesh^18^ found that 82.5% of cases occurred within the first 5 years, with a decrease to 14% in those with up to 10 years of experience and only 3.5% after 10 years. Another study reported that musculoskeletal symptoms often began within the first year of practice (65%).^9^

### WMSDs BY BODY REGION

In Pernambuco, Brazil, manual therapists most frequently reported cervical and lumbar spine disorders (60%), followed by wrist/hand (56%) and shoulder (50%) complaints.^30^ A study found LBP in 44% of physical therapists who did not use armrests, followed by neck and shoulder pain (27% each).^10^ In Kigali, India, the low back was the most affected region (71.1%).^13^

In Malaysia, 70% of participants reported WMSDs, with the low back being the most affected region, followed by the neck.^10^ However, a study found that while LBP was the most prevalent issue, wrist pain also showed notable prevalence (15.5%).^11^ Similar patterns were observed in Ireland, where 49% of therapists reported LBP,^31^ with higher prevalence in women (52%) compared to men (42%)^11^ (Table 3).

**Table 3:** Description of work-related musculoskeletal disorders (WMSDs) by body region and pain^†^, n (ages in %)

First author	Country	Size of study sample (n)	Low back pain* n (%)	Upper back pain n (%)	Neck pain n (%)	Shoulder pain n (%)	Hand pain n (%)	Wrist pain n (%)	Elbow pain n (%)	Hip pain n (%)	Knee pain n (%)	Ankle pain n (%)
Narendrasinh JU^2^	India	60	25 (41.6)	10 (16.6)	16 (26.66)	9 (15.0)	4 (6.66)	4 (6.66)	6 (10.0)	1 (1.66)	3 (5.0)	10 (16.66)
Sagahutu JB^13^	Nigeria	49	38 (77.1)	28 (57.1)	39 (79.0)	25 (51.1)	15 (31.4)	15 (31.4)	3 (5.7)	3 (5.7)	7 (14.6)	0 (0.00)
Anyfantis I^8^	Greece	252	124 (49.0)	63 (25)	48 (19.0)	76 (30.0)	60 (24.0)	60 (24.0)	35 (14.0)	48 (19)	48 (19.0)	—
Balakrishnan R^10^	Malaysia	70	31 (44..0)	10 (14)	14 (20.0)	6 (8.0)	4 (5.0)	4 (5.0)	0	1 (1.0)	8 (11.0)	2 (3.0)
Kutty RK^11^	India	190	70 (36.6)	15 (8)	27 (14.0)	—	13 (7.0)	13 (7.0)	—	10 (5.0)	—	—
Prerana S^14^	India	271	177 (65.3)	43 (15.9)	113 (41.8)	72 (26.5)	43 (15.9)	43 (15.9)	16 (5.9)	11 (4.1)	30 (11.2)	11 (4.1)
Pfumojena CC^15^	Zimbabwe	261	207 (79.3)	135 (51.7)	97 (37.0)	141 (54.0)	109 (41.4)	109 (41.4)	27 (10.3)	15 (5.7)	42 (16.1)	33 (12.6)
Mondal A^16^	Bangladesh	61	45 (28.0)	—	18 (30.0)	8 (12.5)	2 (2.5)	2 (2.5)	—	—	6 (10.0)	—
Nazari H^17^	Hamedan	45	13 (31.7)	7 (17.1)	11 (26.8)	8 (19.5)	14 (34.1)	15 (34.1)	5 (12.2)	3 (7.3)	12 (29.3)	3 (7.3)
Happy SM^18^	Bangladesh	60	43 (71.7)	26 (43.3)	21 (35.0)	8 (13.3)	10 (16.7)	10 (16.7)	3 (6.7)	3 (5.0)	12 (20.0)	9 (15.0)
Iqbal Z^19^	India	75	38 (51.0)	6 (7)	13 (17.0)	9 (12.0)	5 (7.0)	5 (7.0)	11 (15.0)	11 (15.0)	11 (15.0)	11 (15.0)
Vieira ER^9^	United States of America	121	80 (66.0)	43 (35)	74 (61.0)	51 (42.0)	44 (36.0)	44 (36.0)	18 (15.0)	28 (23.0)	44 (36.0)	30 (25.0)
Islam MS^20^	Bangladesh	101	91 (90.0)	82 (82)	73 (72.0)	56 (55.0)	25 (24.0)	25 (24.0)	23 (23)	21 (21.0)	54 (53.0)	46 (45.0)
Muaidi QI^21^	Kingdom of Saudi Arabia	690	321 (46.5)	20 (2.9)	18 (26.5)	84 (12.2)	—	—	70 (10.2)	55 (8.0)	75 (10.9)	24 (3.5)
Abaraogu UO^22^	Nigeria	126	73 (58.0)	38 (29.8)	53 (41.9)	38 (30.1)	36 (28.3)	36 (28.3)	14 (10.8)	21 (16.1)	26 (20.7)	12 (10.0)
Alnaser MZ^23^	Kuwait	312	172 (55.0)	—	34 (11.0)	22 (7.0)	62 (20.0)	62 (20.0)	—	—	—	—
Rahimi F^24^	Iran	319	207 (65.0)	156 (49)	183 (57.4)	160 (50.2)	—	—	69 (21.6)	39 (12.2)	145 (45.5)	63 (19.7)
Ezzatvar Y^25^	Spain	981	485 (49.0)	345 (36.1)	56 (57.0)	332 (33.8)	323 (32.7)	323 (32.7)	164(16.7)	—	—	—
Bae Y-H^26^	South Korea	788	179 (23.0)	179 (22.7)	11 (14.0)	184 (23.3)	179 (22.7)	179 (22.7)	73 (9.3)	63 (8.0)	63 (8.0)	63 (8.0)
Nasir A^27^	Pakistan	56	31 (56.0)	22 (39.5)	35 (62.8)	13 (23.3)	5 (9.3)	5 (9.3)	4 (7.0)	8 (14.0)	7 (11.6)	3 (4.7)

(—) indicates data was not reported in the original study.

* “Low back pain” includes lumbar pain; “hip” includes thigh region.

† Terms such as pain, discomfort, or musculoskeletal pain were all interpreted as pain for consistency.

### JOB-RELATED RISK FACTORS

A total of 19 job-related risk factors were identified across 10 studies (Table 4). Balakrishnan et al.^10^ reported the highest number of contributing factors, while the study by Anyfantis & Biska^8^ found task repetition to be the most frequent factor (90%), followed by working in static postures (82.9%).^9^

**Table 4:** Job-related risk factors contributing to the development of work-related musculoskeletal disorders (WMSDs)

Job risk factors*	Authors									
	Shiba^2^ N = 60, n (%)	Sagahutu^13^ N = 49, n (%)	Anyfantis^8^ N = 252, n (%)	Balakrishnan^10^ N = 70, n (%)	Prerana^14^ N = 271, n (%)	Pfumojena ^15^* n = 261, n (%)	Happy^18^ N = 60, n (%)	Islam^21^** N = 690, n (%)	Alnaser^23^ N = 312, n (%)	Aiza Nasir^27^ N = 60, n (%)
Repetitive tasks	26 (43.3)	5 (11.0)	226 (90.0)	38 (54.3)	132 (48.8)	144 (55.2)	25 (41.7)	277 (40.1)	2 (5.0)	22 (37.2)
Number of patients treated per day	33 (55.0)	15 (31.1)	—	52 (74.3)	185 (68.2)	162 (62.1)	—	209 (30.3)	—	21 (34.9)
Limited breaks during work	33 (55.16)	10 (20.0)	—	-	148 (54.7)	63 (24.1)	42 (70.0)	129 (18.7)	—	27 (44.2)
Manual therapies	32 (53.33)	9 (17.8)	—	48 (68.6)	110 (40.6)	136 (52.2)		204 (29.6)	81 (26.0)	20 (32.6)
Awkward/cramped working posture	24 (40.0)	14 (28.9)	175 (70.0)	43 (61.4)	172 (63.6)	87 (33.3)	14 (23.3)	316 (45.9)	—	20 (39.5)
Reaching/working away from body	29 (48.33)	4 (8.9)	—	39 (55.7)	144 (53.0)	66 (25.3)	—	166 (24.1)	—	8 (14.0)
Back twisting/bending awkwardly	25 (41.66)	14 (28.9)	—	54 (77.1)	178 (65.8)	102 (39.1)	18 (30.0)	129 (18.7)	—	23 (37.2)
Unanticipated/sudden movements	27 (45.0)	3 (6.7)	88 (35.0)	17 (24.3)	128 (47.1)	51 (19.5)	—	171 (24.8)	—	4 (7.0)
Assisting in gait training/activities	28 (46.66)	5 (11.0)	—	33 (47.1)	110 (40.6)	54 (20.7)	—	289 (41.9)	—	12 (18.61)
Lifting/transferring patients	27 (45.0)	15 (31.1)	—	57 (81.4)	155 (57.0)	129 (49.4)	—	0 (0.00)	137 (44.0)	10 (16.3)
Managing agitated/confused patients	25 (41.66)	8 (15.6)	—	28 (40.0)	100 (37.0)	42 (16.1)	—	103 (14.9)	—	4 (7.0)
Carrying/lifting heavy equipment	26 (43.33)	7 (13.3)	—	43 (61.4)	128 (47.1)	45 (17.2)	—	119 (17.2)	—	6 (9.4)
Working at physical limit	23 (38.33)	10 (20.0)	—	42 (60.0)	136 (50.0)	51 (19.5)		235 (34.1)	—	10 (16.3)
Working despite pain/injury	21 (35.0)	13 (26.7)	—	43 (61.4)	173 (63.6)	114 (43.7)	16 (26.7)	143 (20.7)	—	18 (30.2)
Long shifts or overwork	23 (38.33)	4 (8.9)	—	41 (58.6)	150 (55.3)	63 (24.1)	33 (55.0)	216 (31.3)	—	18 (32.6)
Inadequate training for injury prevention	19 (31.66)	8 (15.6)	—	37 (52.9)	134 (49.4)	24 (9.2)	—	136 (19.7)	—	7 (11.6)
Prolonged static posture	—	20 (40.0)	—	58 (82.9)	209 (77.0)	117 (44.8)	36 (60.0)	300 (43.5)	31 (10.0)	—
Significant forces	—	—	76 (30.0)	—	—	—	—	—	—	—
Workplace environmental conditions	—	—	38 (15.0)	—	—	—	—	—	—	—

(—) = data not reported in the original study. N represents the total study population.

* Only major risk factors classified by each author were included.Major or significant risk factors were considered and reported. Other eligible studies did not discuss job-related risk factors.

## DISCUSSION

The aim of this systematic review was to synthesize the current evidence on the prevalence and characteristics of WMSDs among physical therapists WMSDs among physical therapists. According to the World Health Organization, WMSDs encompass a broad range of inflammatory and degenerative conditions that result in pain and functional impairment. Health professionals, particularly those in direct contact with patients, are among the professional groups with the highest rates of WMSDs due to the physical demands of their work and prolonged static or awkward postures throughout the day.^32^

WMSDs are common among health professionals, especially those involved in direct patient care.^33^ Physical therapists perform numerous physically demanding tasks, such as transferring and repositioning patients, frequent trunk flexion and rotation, and maintaining awkward postures. In addition, psychosocial factors have also been linked to the development of WMSDs. Physical therapy involves repetitive tasks, and most physical therapists report symptoms of WMSDs within the first 5 years of practice.^9^

In the current review, the overall prevalence of WMSDs among physical therapists was 75.27%, which is considered high. LBP was the most frequently reported condition (54.97%), followed by neck and upper back pain.^34^ Regarding the upper extremities, the most commonly affected regions were the shoulders and hands/wrists, while the knees were most frequently reported among lower extremity complaints.^11^ However, the prevalence may vary across different specialties.^20^

Although physical therapists are trained in ergonomics and injury prevention, their risk of developing WMSDs remains high. This can result in increased health care costs and reduced productivity. It also may also lead to career changes, decreased clinical longevity, or even premature departure from the profession. Contributing factors to WMSDs include improper biomechanics, repetitive strain, poor flexibility, and muscle overuse. Common outcomes include soft tissue injuries, sprains, strains, spondylosis, herniated discs, osteoarthritis, and neuropathies.^35^

Although LBP is the most prevalent WMSD, it does not necessarily result in absenteeism. Instead, many physical therapists continue working despite symptoms, a phenomenon known as presenteeism. This behavior may reduce work efficiency and increase the likelihood of clinical errors as well as contribute to secondary health issues such as hypertension, arthritis, migraines, insomnia, and mental health disorders. Feelings of fatigue and overload are also associated with job dissatisfaction.^36^

A study reported that 3.1% of physical therapists discontinued work due to aggravated symptoms during patient care.^19^ Pfumojena^15^ observed that approximately 66.7% of WMSDs developed within the first 5-6 years of professional practice,^20,26,37^ although this percentage may vary.^25^ The physical demands placed on the spine — particularly the lumbar region — are especially significant for physical therapists. Consequently, LBP and related symptoms are most prevalent during the early years of professional activity.^13^ However, the frequency tends to decrease after 15 years of experience.^10^ In terms of work experience, Prerana et al.^14^ also found a gradual decline in WMSD prevalence over time.^15^ This trend may be explained by the fact that professional experience enhances ergonomic awareness and the use of self-protection strategies.

Regarding gender, female physical therapists were initially more prone to WMSDs than males, possibly due to lower body mass index (BMI) and physical demands of patient handling.^16,17^ However, with increasing years of experience, the prevalence among male professionals tends to rise, and after 15 years, the gender difference becomes negligible.^14^ Other studies suggest males are ultimately more affected by WMSDs compared to females.^21^

In relation to manual therapy, a study involving 361 physical therapists reported that the most affected regions were the wrists and hands (80.6%), followed by the back (28%).^38^ Concerning BMI, 64% of females were in the normal weight range, while 53.8% of males and 23.2% of females were overweight, and 15.2% of males and 11.6% of females were obese. These factors may negatively impact personal and family life, including physical fitness, leisure activities, and daily functioning.

Another study involving 1,232 health professionals indicated that WMSDs may be associated with moderate to high levels of occupational stress, which could contribute to sleep disturbances. However, the association between WMSDs and sleep disorders was found to be weak.^39^

Regarding ergonomic risks, 224 clinical physical therapy sessions performed by 29 physical therapists were analyzed. Among the 224 observations (132 adult and 92 pediatric sessions), physical therapists were frequently found working in sitting, standing, or kneeling postures, with 82.59% of tasks classified as presenting high or very high ergonomic risk. Pediatric sessions were associated with higher ergonomic risk than adult sessions.^40^

With regard to WMSD management strategies, physical therapists typically seek medication, consult physicians, and modify patient-handling techniques or body positioning. Preventive strategies to reduce the incidence of WMSDs among physical therapists include limiting the number of patients seen per day in clinical settings; incorporating scheduled breaks during work shifts; reinforcing proper ergonomics through ongoing training, especially in manual therapy settings; implementing assistive devices to standardize therapist posture; conducting annual health screenings in rehabilitation centers and physical therapy departments; and encouraging daily physical activity to maintain general fitness. Finally, the limitations of this study were the use of only two search engines and the time frame from 2015 to May 30, 2021. Nevertheless, we believe this review offers valuable insights for the development of future protocols aimed at preventing the occurrence of WMSDs among physical therapists.

## CONCLUSIONS

Physical therapists had a high prevalence of WMSDs. The most commonly affected body region was the lower back, followed by the neck and wrist. Male professionals were more frequently affected, and the injuries were primarily associated with ergonomic risk factors and individual physical conditions — especially among those with 1-5 years of professional experience. Most of the included studies were cross-sectional, which highlights the need for longitudinal research to better understand how the prevalence of WMSDs evolves over time.
